# Cardiovascular and Renal Disease in Chronic Critical Illness

**DOI:** 10.3390/jcm10081601

**Published:** 2021-04-09

**Authors:** Tyler J. Loftus, Amanda C. Filiberto, Tezcan Ozrazgat-Baslanti, Saraswathi Gopal, Azra Bihorac

**Affiliations:** 1 Department of Surgery, University of Florida Health, Gainesville, FL 32603, USA; amanda.filiberto@surgery.ufl.edu; 2 Precision and Intelligent Systems in Medicine (PrismaP), College of Medicine, University of Florida, Gainesville, FL 32611, USA; tezcan.ozrazgatbaslanti@medicine.ufl.edu (T.O.-B.); abihorac@ufl.edu (A.B.); 3 Sepsis and Critical Illness Research Center, University of Florida Health, Gainesville, FL 32610, USA; 4 Department of Medicine, University of Florida Health, Gainesville, FL 32603, USA; saraswathi.gopal@medicine.ufl.edu

**Keywords:** critical care, intensive care unit, acute kidney injury, chronic kidney disease, heart failure

## Abstract

With advances in critical care, patients who would have succumbed in previous eras now survive through hospital discharge. Many survivors suffer from chronic organ dysfunction and induced frailty, representing an emerging chronic critical illness (CCI) phenotype. Persistent and worsening cardiovascular and renal disease are primary drivers of the CCI phenotype and have pathophysiologic synergy, potentiating one another and generating a downward spiral of worsening disease and clinical outcomes manifest as cardio-renal syndromes. In addition to pharmacologic therapies (e.g., diuretics, beta adrenergic receptor blockers, angiotensin-converting enzyme inhibitors, angiotensin II receptor blockers, and blood pressure control), special consideration should be given to behavioral modifications that avoid the pitfalls of polypharmacy and suboptimal renal and hepatic dosing, to which CCI patients may be particularly vulnerable. Smoking cessation, dietary modifications (e.g., early high-protein nutrition and late low-sodium diets), and increased physical activity are advised. Select patients benefit from cardiac re-synchronization therapy or renal replacement therapy. Coordinated, patient-centered care bundles may improve compliance with standards of care and patient outcomes. Given the complex, heterogeneous nature of cardiovascular and renal disease in CCI and the dismal long-term outcomes, further research is needed to clarify pathophysiologic mechanisms of cardio-renal syndromes in CCI and develop targeted therapies.

## 1. Introduction

Advances in critical care diagnostics, treatments, and organizational structures have decreased intensive care unit (ICU) and in-hospital mortality, despite increasing illness severity [1,2,3,4]. Patients with multiple organ failure and long ICU stays, who would have succumbed to illness in previous eras, now survive through hospital discharge. Unfortunately, many of these survivors suffer from chronic organ dysfunction and induced frailty, representing an emerging chronic critical illness (CCI) phenotype with poor long-term outcomes and increased healthcare resource use [5,6]. Persistent and worsening cardiovascular and renal disease are primary drivers of the CCI phenotype [7,8]. Cardiovascular and renal disease appear to have pathophysiologic synergy in which renal disease potentiates cardiovascular disease and cardiovascular disease potentiates renal disease, creating a cycle of worsening disease burden and clinical outcomes manifest as cardio-renal syndromes [9,10,11,12,13]. Early identification and optimal treatment of cardiovascular and renal disease can optimize patient outcomes and resource use among ICU and CCI patients. This article defines the epidemiology of cardiovascular and renal disease in CCI, summarizes salient pathophysiology, and describes targeted therapies.

## 2. Definitions and Epidemiology of Chronic Critical Illness

First described by Girard and Raffin, [5] CCI is now variably defined as a disease state characterized by prolonged (8–14 days or more) ICU stay with persistent organ dysfunction [6,14,15,16]. The onset of CCI can be conceptualized as the point at which patient demographics and chronic disease burden become the strongest predictors of clinical outcomes. Using this approach, Iwashyna et al. [16] demonstrated that the onset of critical illness usually occurs approximately 10 days after ICU admission, and ranges from 7–22 days across various primary diagnosis categories. 

Chronic critical illness is common and is associated with poor long-term outcomes and high resource use. Prolonged mechanical ventilation and sepsis are the most common etiologies [6]. The incidence of CCI among ICU patients is approximately 5–8%; these patients account for more than 30% of all ICU resource use [6,16]. In the United States alone, there are more than 380,000 cases of CCI per year, accounting for approximately $26 billion in hospital-related expenditures [6]. The incidence of CCI varies by primary diagnosis. After ICU admission for severe blunt trauma, CCI develops in approximately 20% of all patients; after ICU admission for sepsis, CCI develops in approximately 50% of all patients [17,18]. The overall chronic disease burden associated with CCI is substantial. Six months after discharge, approximately two-thirds of all CCI survivors have too much cognitive impairment to perform cognitive assessments by phone [19]. One year after discharge, only about 10% of all CCI patients live independently [20]. Due to the high incidence of CCI and dismal long-term outcomes, improving care for CCI patients should be a high priority for critical care clinicians, researchers, and policymakers. 

## 3. Definitions, Epidemiology, and Risk for Cardiovascular and Renal Disease in Chronic Critical Illness

### 3.1. Cardiovascular Disease

Cardiovascular disease is a primary driver of mortality, morbidity, and healthcare costs. In 2009, cardiovascular disease accounted for approximately one in six hospital admissions, including six million admissions in the United States alone [21]. These admissions cost over $70 billion, or one quarter of all inpatient hospital costs. Inpatient mortality during admission for cardiovascular disease was 3.2%, compared with 2.1% for all other causes for admission. CCI may intensify the clinical and financial burdens of cardiovascular disease. Chronic cardiovascular disease is difficult to define and recognize in research and administrative databases due to the heterogeneous nature of heart muscle, valve, arterial, and conduction system diseases. Instead, heart failure can serve as a surrogate for chronic cardiovascular disease. In a study of 17,478 patients who survived more than 30 days after ICU admission, the overall incidence of congestive heart failure was 25%, and was significantly greater (46%) among subjects who expired within the following year [7]. Subjects who expired within one year also had greater acute requirements for cardiovascular support, manifested as significantly longer duration of vasopressor use (median 1.5 days vs. 0.8 days). These observations suggest that acute cardiovascular dysfunction during critical illness is associated with chronic cardiovascular disease and worse long-term outcomes, consistent with the CCI phenotype.

### 3.2. Renal Disease

Similar to acute and chronic cardiovascular disease, acute and chronic kidney disease have substantial negative impacts on clinical outcomes and healthcare expenditures. Acute kidney injury (AKI), defined as an abrupt decrease in kidney function over seven days or less, occurs in 7–18% of all hospitalized patients, and approximately half of all ICU patients [22,23,24]. AKI accounts for more than two million deaths worldwide each year [25]. AKI survivors are at increased risk for developing chronic kidney disease (CKD), characterized by abnormal kidney structure or function persisting for more than 90 days, and end-stage renal disease (ESRD), characterized by requirements for dialysis or kidney transplantation [22]. The risk of developing ESRD is 13 times greater among patients with AKI versus no AKI; the risk of ESRD is 40 times greater among patients with both AKI and CKD [26]. Among critically ill patients with AKI requiring renal replacement therapy, in-hospital mortality is 50–80% [27]. AKI progresses along a spectrum of disease to CKD and ESRD, with worsening outcomes and financial burdens with each phase of disease progression.

Recent expert-derived classification systems provide greater granularity in describing the spectrum of acute and chronic kidney disease. The 16th Acute Disease Quality Initiative (ADQI) proposed distinct clinical trajectories of kidney injury, including rapidly reversed (within 48 h) AKI, persistent AKI (beyond seven days, denoting acute kidney disease) with recovery of baseline renal function within 90 days, and persistent AKI without recovery of baseline renal function [8]. These trajectories and their associated clinical phenotypes suggest that management strategies targeting early reversal of AKI and recovery of renal function have the potential to mitigate the adverse short- and long-term consequences of kidney disease progression. Further research is needed to define renal recovery and apply this more granular ADQI classification system for diagnostic and therapeutic prognostication and decision-making.

Similar to the observation that acute cardiovascular dysfunction during critical illness is associated with chronic cardiovascular disease and worse long-term outcomes, CCI appears to affect kidney injury trajectories adversely. Among subjects who survive more than 30 days after ICU admission, the overall incidence of ESRD was 1.1%, and was significantly greater (3.2%) among subjects who expired within one year [7]. Subjects who expired within one year also had greater incidence of AKI (18.3% vs. 6.3%), mimicking the acute and persistent chronic disease paradigm observed for cardiovascular disease. Among ICU patients, early reversal of AKI and recovery of renal function are viable strategies for improving short- and long-term clinical outcomes. 

The duration and severity of AKI for sepsis patients has important implications for long-term survival and physical function. In a prospective observational study of 239 patients with surgical sepsis, the overall incidence of AKI was 62% [28]. AKI persisted for three days or more in approximately two-thirds of these patients, conferring a 3-fold increased risk for 30-day and 1-year mortality. Only 57% of all patients with persistent AKI were alive one year after discharge. Among survivors, only 12% could perform pre-disease activities without restrictions. Biomarker analyses suggested that persistent AKI patients had more profound early physiological derangement as well as early and persistent immune dysregulation and endothelial dysfunction. Collectively, these findings suggest that early reversal of AKI is critically important to achieving optimal short- and long-term patient outcomes and functional recovery, and that immune and endothelial dysfunction may represent early therapeutic targets. Further research is needed to develop and validate targeted therapies to prevent and treat AKI and persistent AKI among sepsis patients.

### 3.3. Associations between Renal and Cardiovascular Disease 

Renal and cardiovascular disease have pathophysiologic synergy, i.e., renal disease potentiates cardiovascular disease and cardiovascular disease potentiates renal disease, creating a cycle of worsening disease burden and clinical outcomes manifest as cardio-renal syndromes [9,12,13]. Once glomerular filtration rate is less than 75 mL/min per 1.73 m^2^, risk for cardiovascular mortality increases linearly as GFR decreases linearly, while adjusting for potentially confounding cardiovascular risk factors (e.g., hypertension and diabetes) and albuminuria [10,29,30]. Compared with subjects who have normal renal function, stage 3 CKD is associated with two-fold increased cardiovascular mortality; stage 4 CKD is associated with three-fold increased cardiovascular mortality. The cardiovascular diseases implicated in these risk assessments include coronary artery disease, heart failure, atrial fibrillation, peripheral arterial disease, and stroke; risk for each of these conditions is roughly doubled for patients with stage 3 CKD [31,32,33,34,35]. Among patients with stage 3 CKD, the incidence of cardiovascular mortality is even greater than the incidence of kidney failure requiring renal replacement therapy [10,29,36]. Cardiovascular disease also increases risk for renal disease, primarily due to renal hypoperfusion in heart failure with reduced ejection fraction [37]. In addition, atherosclerotic renal arterial stenosis decreases afferent arteriolar blood flow and precludes the use of angiotensin-converting enzyme inhibitors and angiotensin receptor blockers, which are associated with efferent arteriolar dilation and decreased glomerular filtration rate [11]. Collectively, these findings demonstrate that kidney disease independently and substantially increases risk for adverse cardiovascular outcomes, and that cardiovascular disease increases risk for renal disease. The pathophysiologic mechanisms driving these clinical manifestations are complex.

## 4. Pathophysiology of Cardiovascular and Renal Disease in Chronic Critical Illness

**Figure 1:**
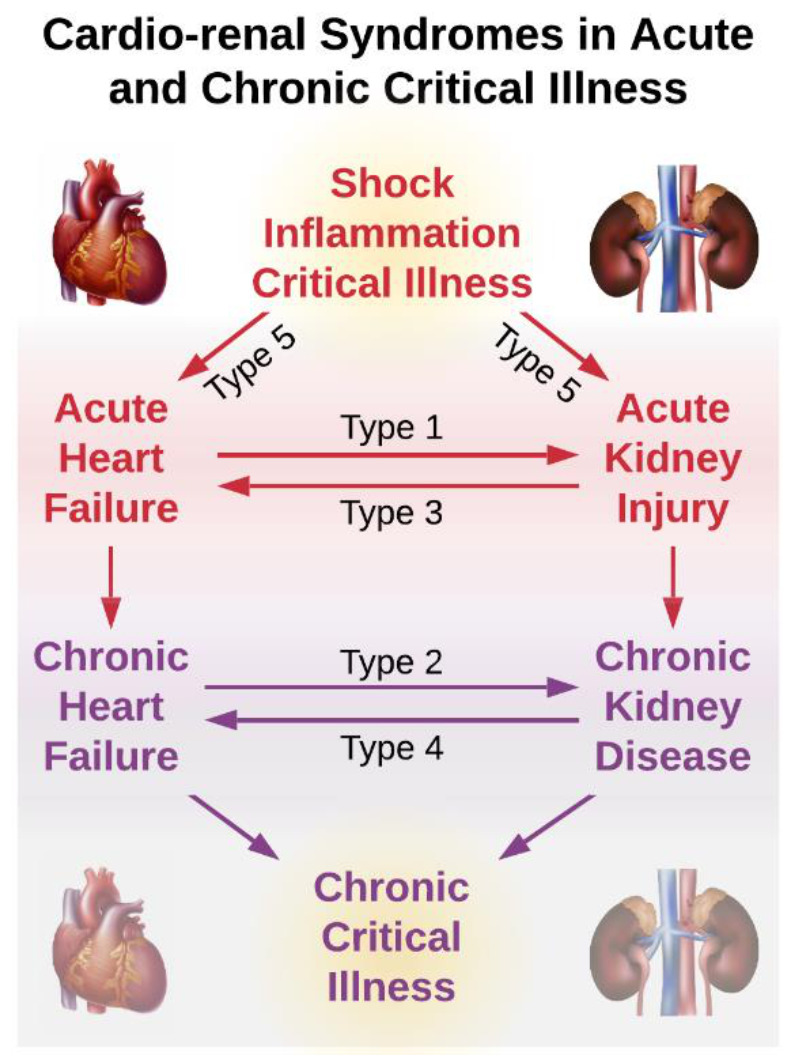
After an acute physiologic insult, acute heart and kidney disease lead to chronic heart and kidney disease and chronic critical illness. Cardio-renal syndrome type 1: acute heart failure precedes acute kidney injury; type 2: chronic heart failure precedes chronic kidney disease; type 3: acute kidney injury precedes acute heart failure; type 4: chronic kidney disease precedes chronic heart failure; type 5: acute shock, inflammation, or critical illness precede acute heart failure or kidney injury.

Cardiovascular and renal disease appear to have pathophysiologic synergy in which renal disease potentiates cardiovascular disease and cardiovascular disease potentiates renal disease, creating a cycle of worsening disease burden and clinical outcomes [9,10,11]. These cardio-renal syndrome pathways are bi-directional, i.e., when cardiac disease causes renal disease, worsening renal disease in turn causes worsening heart disease, and vice versa [12,13]. In addition, the inciting event for acute heart failure or AKI can be shock, inflammation, and critical illness, which can lead to CCI, potentiating this cycle. Associations among cardio-renal syndromes and acute and chronic phases of critical illness are illustrated in Figure 1.

There are five types of cardio-renal syndrome [12,13]. Types one, two, three, and four involve acute or chronic cardiac disease causing renal disease, and acute or chronic renal disease causing heart disease. The pathways by which renal disease causes or worsens cardiovascular disease are primarily related to left ventricular hypertrophy and reduced cardiac perfusion. Several features of CKD are associated with left ventricular hypertrophy and reduced coronary reserve: hypertension, renal anemia, arteriosclerosis, reduced cardiac capillary density, and impaired coronary vasodilation in the context of decreased endothelial expression of nitric oxide synthase [38,39,40,41]. Left ventricular hypertrophy involves myocardial fibrosis, which impairs contractility and is associated with increased risk for dysrhythmias and sudden cardiac death [42,43]. This, along with electrolyte imbalances and accelerated coronary artery disease associated with pro-inflammatory, atherogenic lipid profiles, may explain why the incidence of sudden cardiac death is increased among patients with kidney failure. Sudden cardiac death accounts for 26% of all deaths among patients with kidney failure, while accounting for only 6–13% of all deaths in the general population [43,44]. When new or worsening cardiovascular disease leads to heart failure with reduced ejection fraction, renal function may be further impaired by renal hypoperfusion [37]. Therefore, renal disease has direct, negative impacts on cardiovascular function, increasing risk for death due to cardiovascular disease, and impaired cardiac output can reduce renal blood flow, worsening the cycle of disease.

In type five cardio-renal syndrome, the inciting pathology is a systemic disease such as severe acute inflammation or shock [12,13]. Sepsis, defined as a dysregulated host response to infection leading to life-threatening organ dysfunction, is a common etiology of type five cardio-renal syndrome. Sepsis is a common cause of critical illness, a common secondary complication among ICU patients, and is associated with 18–28% mortality [45,46,47]. Sepsis is also uniquely associated with cardiovascular and renal disease. More than half of all septic patients develop AKI [48,49]. Renal injury among septic patients is often persistent. More than 60% of all CCI patients had AKI during the acute phase of their illness, and non-recovery from AKI portends dismal long-term outcomes [50,51,52,53]. Cardiac disease is also common in sepsis. Troponin elevations occur in as many as 70–80% of all patients with sepsis and septic shock [54,55,56]. In septic patients, troponin elevations are associated with decreased left ventricular function [57,58]. In a prospective, observational study of critically ill patients with sepsis or septic shock who underwent echocardiography early after ICU admission, the incidence of diastolic dysfunction, defined as e′-wave <8 cm/s with left ventricular ejection fraction >50%, was 40.4%; diastolic dysfunction was associated with increased mortality (hazard ratio 6.0) relative to preserved systolic and diastolic dysfunction [14]. Once cardiac or renal disease is instigated by sepsis, there is increased risk for bi-directional, synergistic worsening cardiac and renal disease [12,13]. For example, even in cases of preserved ejection fraction, longstanding diastolic heart failure can worsen renal function via chronic hypertension and increased central venous pressure as well as endothelial dysfunction and dysregulation of the renin-angiotensin aldosterone system [15].

Chronic critical illness can also predispose to persistent cardiovascular and renal disease by inducing frailty, or loss of physiologic reserve and increased vulnerability to physiologic stressors [59]. This may manifest as increased healthcare resource use after critical illness. In a large cohort of ICU survivors, almost 50% were readmitted to the hospital within one year of discharge, demonstrating a predilection for presentation to emergency department and inpatient services rather than to outpatient primary care services [60]. The pathophysiology of accumulating chronic disease burden and induced frailty after CCI is complex. The persistent inflammation, immunosuppression, and catabolism syndrome (PICS) offers biologically plausible explanations [14,61]. In a subset of CCI patients, acute inflammation persists, and is accompanied by elaboration of myeloid-derived suppressor cells, which are immunosuppressive, immature myeloid cells [14,61]. PICS patients also have muscle catabolism despite adequate nutrition, demonstrated by a loss of up to 30% of all lean body mass within weeks of the acute inflammatory insult [62,63]. Muscle biopsy of CCI survivors demonstrates defective mitochondria, myocyte necrosis, and leukocyte infiltration, implicating inflammation and mitochondrial damage in catabolism after CCI [64]. New or ongoing AKI leaking to CKD appears to instigate PICS through catabolic and pro-inflammatory pathways [65]. Together, these findings suggest that CCI leading to PICS is associated with global inflammatory, immunosuppressive, and catabolic changes that potentiate organ dysfunction.

## 5. Predicting Cardiovascular and Renal Disease in Chronic Critical Illness

Accurately predicting risk for cardiovascular and renal disease has the potential to inform prognostic discussions with patients and augment clinical decision-making. It may be useful to identify high-risk patients that benefit from targeted resource-intense management strategies and low-risk patients for whom intensity and frequency of surveillance and diagnostic testing can be safely decreased. Predicting cardiovascular disease is especially important because subjects with diabetic visceral neuropathy often have atypical or asymptomatic ischemic heart disease leading to delayed or missed diagnosis. Not surprisingly, subjects with CKD have worse prognoses for dysrhythmias and myocardial infarctions compared with subjects that do not have CKD [66,67]. In addition, the sensitivity of cardiac troponins for detecting myocardial infarction is decreased among patients with CKD, partly due to greater baseline troponin values and impaired troponin clearance [68]. Therefore, using other methods to predict adverse cardiovascular events has the potential to improve prognostication and augment clinical decision-making by avoiding preventable harm from missed diagnoses.

AKI is common among hospitalized chronically ill patients, yet there are no widely accepted models for predicting risk of subsequent CKD. James et al. [69] developed a multivariable model that included age, sex, acute kidney injury stage, prehospitalization serum creatinine, albuminuria, and discharge creatinine that achieved a c statistic of 0.81 (0.75–0.86) in predicting post-discharge advanced CKD. Data readily obtainable from electronic health records (EHRs) could be used to derive and validate models that predict risk for developing CKD among patients with chronic critical illness. In a systematic review of 212 studies evaluating 363 models predicting risk for cardiovascular disease, only 132 studies were externally validated and model performance measures were heterogenous, hindering direct comparisons of model performance [70]. There are abundant models predicting incident cardiovascular disease in the general population. In this era of large datasets, studies should aim to validate existing models, improve them by adding new predictors or more observations, and tailor models to high-risk populations and risk-sensitive decisions. 

To date, there are no published models predicting CKD or cardiovascular disease specifically among chronic critical illness patients. However, with the widespread availability of large volumes of EHR data and validated models predicting CKD and cardiovascular disease in the general population, there is a unique opportunity to more precisely classify and predict cardiovascular and renal outcomes. In 2009, the Health Information Technology for Economic and Clinical Health Act of 2009 incentivized EHR adoption [71]. Six years later, more than 80% of all US hospitals had adopted EHRs, producing massive amounts of data, which accumulate at increasing rates each year [72,73]. Lysak et al. [74] leveraged EHR data to predict cardiovascular-specific mortality and progression to ESRD among surgical patients with good accuracy (c-statistics of 0.77 and 0.82, respectively). A similar approach could be used to predict cardiovascular and renal outcomes after CCI; this information could augment discussions regarding prognosis and decisions regarding resource use.

## 6. Therapeutic Interventions for Cardiovascular and Renal Disease in Chronic Critical Illness

There is a paucity of literature investigating the efficacy of therapeutic interventions for cardiovascular and renal disease among CCI patients. Therefore, this section focuses primarily on evidence for the treatment of chronic cardiovascular and renal disease, and seeks to apply this evidence within the CCI paradigm.

### 6.1. Behavioral and Lifestyle Interventions

In treating and preventing the progression of cardiovascular and renal disease, special consideration should be given to behavioral and lifestyle interventions. This approach is cost-neutral and avoids polypharmacy in a CCI population that may have dynamic impairments in renal and hepatic function that necessitate precise dosing. For example, smoking cessation is medically advantageous for anyone, especially those affected by cardiovascular disease, renal disease, or CCI. Associations between smoking and cardiovascular disease are exaggerated among subjects with comorbid CKD [75]. In addition, smoking is associated with progression of CKD [75]. Although there is a lack of high-level evidence that smoking cessation decreases mortality among these subjects, it is reasonable to assume that the well-documented benefits of smoking cessation among general populations apply to subjects with cardiovascular and renal disease. Similar to smoking cessation, the universal benefits of exercise may be even greater among subjects with cardiovascular disease, renal disease, and CCI. Increased physical activity is associated with decreased all-cause and cardiovascular mortality among patients with CKD as well as decreased albuminuria [76,77,78]. Completing an average of 15 min of moderate intensity exercise per day is associated with a 13% decrease in all-cause mortality; each additional 15 min of exercise per day reduces all-cause mortality by 4% [76]. Therefore, smoking cessation and daily exercise may be especially beneficial for subjects with CCI. 

Optimal approaches to nutritional therapy for CCI patients depend on severity of illness, comorbid conditions, and phases of care. Early in critical illness, high-protein diets that maintain positive nitrogen balance without overfeeding have been associated with decreased mortality in observational studies [79,80,81,82]. The benefits of early, high-protein nutrition are greatest for subjects with high risk for adverse events related to malnutrition. These subjects can be identified as those with a Nutrition Risk in the Critically Ill score greater than five [82,83]. Given the catabolic nature of CCI, protein intake of 1.5 g/kg/day or greater is considered adequate by many experts [15,81,84]. These recommendations are primarily derived from observational studies of hospitalized patients; it is unclear whether high-protein diets are effective during post-discharge phases of care. In post-discharge phases, patients with CKD may benefit from low-sodium diets with normal protein (i.e., 0.8 g/kg/day) intake [85,86]. Collectively, these findings suggest that smoking cessation, physical activity, and dietary modifications offer effective, cost-neutral methods for improving clinical outcomes among CCI patients, especially those with CKD.

### 6.2. Pharmacologic Interventions

Pharmacologic interventions for CCI patients should be targeted to specific cardiovascular and renal disease states. Pharmacologic treatment options are listed in Table 1.

Of the many pharmacologic interventions that have been tested for efficacy in managing renal and cardiovascular disease in CCI or similar populations, only diuretic therapy, beta-adrenergic receptor blockers, angiotensin-converting enzyme inhibitors, angiotensin II receptor blockers, and other medications targeting blood pressure control have had consistent, positive results in clinical trials. Data from randomized trials demonstrate that among patients with both diabetic and non-diabetic renal disease, inhibition of the renin-angiotensin aldosterone system with angiotensin-converting enzyme inhibitors or angiotensin II receptor blockers to reduce albuminuria and systolic blood pressure is associated with cardiovascular protection and improved prognosis [87,88]. For the best outcomes, albuminuria should be reduced by at least 50% [88,89]. Although blood pressure targets of less than 140/90 mmHg are sufficient for reducing risk for cardiovascular disease progression in general populations, blood pressure targets of less than 130/80 mmHg are preferable for patients with CKD and albuminuria [88,90]. For critically ill trauma patients, erythropoietin therapy has been shown to increase hemoglobin concentrations and decrease 29-day mortality [16]. Epidemiologic studies suggest that hyperphosphatemia is associated with increased morbidity and mortality, primarily due to cardiovascular disease [91,92]. These observations may be attributable to associations between hyperphosphatemia and arteriosclerosis, suggesting that controlling phosphate levels could mitigate risk for cardiovascular morbidity and mortality [93]. However, in a large randomized trial, the calcimimetic agent cinacalcet did not significantly decrease risk of death or major cardiovascular events among dialysis patients with moderate-severe secondary hyperparathyroidism [94]. Other novel phosphate binders and calcium and vitamin D supplements should be considered for patients with hyperparathyroidism secondary to CKD [17,18]. Sodium-glucose contransporter-2 inhibitors can prevent cardiovascular events and progression to end-stage renal disease among patients with type 2 diabetes [19]. Angiotensin receptor-nephrilysin inhibitors have demonstrated superiority over angiotensin converting enzyme inhibitor therapy alone in decreasing risk for death and hospitalization for heart failure, without increasing adverse events [20,21]. These effects may be attributable to the ability of nephrilysin inhibitors to increase blood levels of endogenous natriuretic peptides. For patients with cardiovascular disease, optimal medical management is specific to the nature of disease (e.g., heart failure, valvular disease, coronary artery disease, dysrhythmias, etc.), and is beyond the scope of this review. In cases of heart failure with reduced ejection fraction, which compromise renal perfusion, medical management should include diuretic therapy, an angiotensin-converting enzyme inhibitors or angiotensin II receptor blocker, and beta-adrenergic receptor blockade. This approach is associated with slowing or reversal of left ventricular hypertrophy, improved symptoms, and increased survival [95,96].

### 6.3. Procedural Interventions

Cardiac re-synchronization and renal replacement therapy are beneficial for select populations of CCI patients. In general, behavioral and lifestyle modifications and pharmacologic interventions should be optimized before implementing procedural interventions. For patients with symptomatic heart failure and decreased ejection fraction or QRS prolongation (>120 ms), cardiac re-synchronization therapy (i.e., placement of a cardiac defibrillator or pacemaker) is indicated to improve symptoms, reduce hospitalization, and reduce mortality [97]. If this approach fails, left ventricular assist device placement and heart transplantation should be considered [98]. Although hyperparathyroidism secondary to CKD usually responds to medical therapy, each year, approximately 1–2% of all patients with secondary hyperparathyroidism undergo parathyroidectomy for refractory disease [22]. Patients with concomitant heart and renal failure are often hypervolemic, requiring intensive, high-dose diuretic therapy. In cases of or hypervolemia that is refractory to diuretic therapy or severe metabolic derangement, renal replacement therapy may be required. Other indications for renal replacement therapy among patients with advanced kidney disease are beyond the scope of this review; interested readers are referred to reviews by Bagshaw, Himmelfarb, and others [99,100]. Although early renal replacement therapy with continuous hemofiltration and hemodialysis techniques have the theoretical advantages of early normalization of metabolic derangements and volume overload, the optimal timing and technique for renal replacement therapy among critically ill patients with cardio-renal syndromes have not yet been established [23].

### 6.4. Treatment Paradigms

Given the substantial burden of mortality, morbidity, and healthcare costs associated with CCI, it is essential to provide effective treatments to this population of patients. Unfortunately, standard care established by clinical practice guidelines, society recommendations, and high-level evidence is often not provided to patients. Compared with the general population, CKD patients with myocardial infarction are less likely to receive counseling regarding smoking cessation, exercise, and weight loss, and receive fewer prescriptions for aspirin, clopidogrel, beta-blockers, and statins [101]. Whether this disturbing phenomenon is due to patient factors or therapeutic nihilism among clinicians is unclear. Regardless, effective methods for delivering standard care to CCI patients are needed. A coordinated, multi-modal, patient-centered approach may yield the best outcomes [86,102,103]. This approach has been associated with an approximately 50% reduction in long-term cardiovascular mortality [104]. Therefore, it seems prudent to incorporate bundles of therapies with established efficacy in patient-centered treatment paradigms for CCI patients.

### 6.5. Acute Kidney Injury (AKI) Survivor Follow-Up Pathways

**Figure 2:**
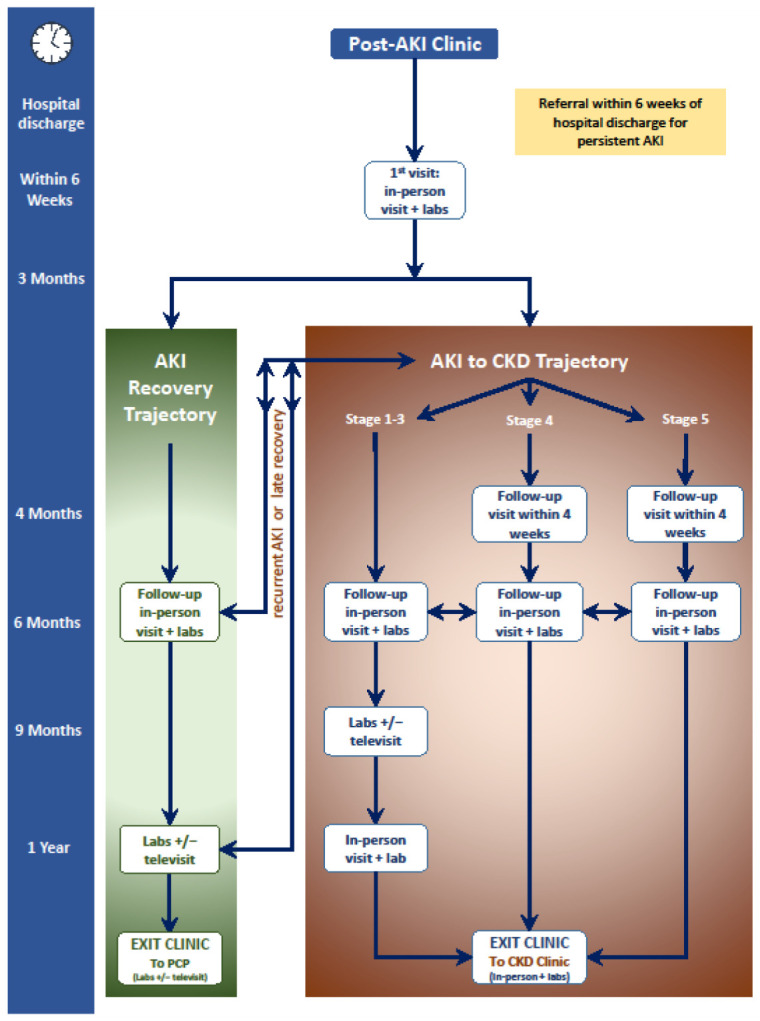
Timeline and workflow for a post-acute kidney injury (AKI) clinic. PCP: primary care provider; CKD: chronic kidney disease.

Patients who develop AKI during hospital admission have a 10-fold greater risk of de novo CKD, a 3-fold greater risk of end-stage renal disease (ESRD), and double the risk of death, compared with patients who do not develop AKI during hospital admission [105]. AKI is also associated with increased long-term incidence of stroke and cardiovascular disease [106,107,108,109]. Despite substantial evidence that AKI is associated with poor long-term outcomes manifest as both renal and non-renal sequelae, there is no current, published standard for follow-up of AKI survivors by nephrologists. Evidence regarding post AKI follow-up care is also limited. The expert consensus group ADQI (Acute Dialysis Quality Initiative) has recognized the need for specialized care for AKI survivors. Based on ADQI guidelines, the authors propose a model for post-AKI follow-up, illustrated in Figure 2 [110].

The focus of AKI survivor clinics is preservation of renal function and preventing further episodes of AKI by modifying risk factors. This includes management of hypertension, proteinuria, metabolic complications associated with AKI or progressing CKD, avoiding nephrotoxins, and patient education.

## 7. Conclusions

Advances in critical care have led to improved survival for critically ill patients. Unfortunately, ICU survivors often suffer from persistent organ dysfunction and poor long-term outcomes and quality of life. Cardiovascular and renal disease are primary drivers of CCI and have pathophysiologic synergy, potentiating one another and generating a downward spiral of worsening disease and clinical outcomes manifest as cardio-renal syndromes. In addition to proven pharmacologic therapies (e.g., diuretic therapy, beta adrenergic receptor blockers, angiotensin-converting enzyme inhibitors, angiotensin II receptor blockers, and other medications targeting blood pressure control), special consideration should be given to effective, cost-neutral behavioral modifications that avoid the pitfalls of polypharmacy and suboptimal renal and hepatic dosing, to which CCI patients may be particularly vulnerable. Smoking cessation, dietary modifications (e.g., early high-protein nutrition and late low-sodium diets), and increased physical activity are advised. Certain patients with progressive cardiac or renal disease despite behavioral modifications and optimal medical management may benefit from cardiac re-synchronization therapy or renal replacement therapy. Coordinated, patient-centered care bundles may improve compliance with standards of care and patient outcomes. Given the complex, heterogeneous nature of cardiovascular and renal disease in CCI and the dismal long-term effects on patient outcomes and healthcare resource use, further research is needed to more precisely elucidate pathophysiologic mechanisms of cardio-renal syndromes in CCI and develop targeted therapies.

## Figures and Tables

**Table 1:** Pharmacologic interventions for cardiovascular and renal disease in chronic critical illness.

Medication	Application for Cardiovascular and Renal Disease in Chronic Critical Illness
Angiotensin II receptor blockers	Reduce albuminuria by at least 50%, reduce systolic blood pressure to less than 140/90 mmHg for general populations and less than 130/80 mmHg for patients with chronic kidney disease and albuminuria
Angiotensin receptor-nephrilysin inhibitors	Reduce albuminuria and blood pressure while increasing blood levels of endogenous natriuretic peptides
Angiotensin-converting enzyme inhibitors	Reduce albuminuria by at least 50%, reduce systolic blood pressure to less than 140/90 mmHg for general populations and less than 130/80 mmHg for patients with chronic kidney disease and albuminuria
Beta-adrenergic receptor blockers	Decrease heart rate, dysrhythmias, and beta-adrenergic receptor overstimulation among patients with chronic left ventricular dysfunction
Calcium and vitamin D supplements	Abrogate hypocalcemia among patients with chronic kidney disease and secondary hyperparathyroidism
Diuretic therapy	Optimize preload and restore normal interstitial water volumes while maintaining intravascular euvolemia
Erythropoietin	Increase hemoglobin levels during critical illness; decrease mortality among critically ill trauma patients
Phosphate binders	Abrogate hyperphospatemia among patients with chronic kidney disease and secondary hyperparathyroidism
Sodium-glucose contransporter-2 inhibitors	Prevent cardiovascular and renal disease and progression among patients with type 2 diabetes

## Data Availability

No new data were created or analyzed in this study. Data sharing is not applicable to this article.
